# Implementation of patient-reported outcomes for symptom management in oncology practice through the SIMPRO research consortium: a protocol for a pragmatic type II hybrid effectiveness-implementation multi-center cluster-randomized stepped wedge trial

**DOI:** 10.1186/s13063-022-06435-1

**Published:** 2022-06-16

**Authors:** Michael J. Hassett, Sandra Wong, Raymond U. Osarogiagbon, Jessica Bian, Don S. Dizon, Hannah Hazard Jenkins, Hajime Uno, Christine Cronin, Deborah Schrag, SIMPRO Co-Investigators

**Affiliations:** 1 Dana-Farber Cancer Institute/Brigham and Women’s Hospital, 450 Brookline Avenue, Boston, MA 02215 USA; 2 Dartmouth-Hitchcock Medical Center, Lebanon, NH USA; 3 Baptist Medical Center, Memphis, TN USA; 4 Maine Medical Center, Portland, ME USA; 5 Lifespan Cancer Institute and Brown University, Providence, RI USA; 6 West Virginia University Cancer Center, Morgantown, WV USA; 7 Memorial Sloan Kettering Cancer Center, New York, NY USA

**Keywords:** Electronic health record, Electronic medical record, Patient-reported outcomes (PROs), Patient-reported outcomes measures (PROMS), Patient Reported Outcomes version of the Common Terminology Criteria for Adverse Events (PRO-CTCAE®), Symptom management, Gastrointestinal cancers, Gynecologic cancers, Thoracic cancers, Chemotherapy, Surgery, Pragmatic clinical trial design

## Abstract

**Background:**

Many cancer patients experience high symptom burden. Healthcare in the USA is reactive, not proactive, and doctor-patient communication is often suboptimal. As a result, symptomatic patients may suffer between clinic visits. In research settings, systematic assessment of electronic patient-reported outcomes (ePROs), coupled with clinical responses to severe symptoms, has eased this symptom burden, improved health-related quality of life, reduced acute care needs, and extended survival. Implementing ePRO-based symptom management programs in routine care is challenging. To study methods to overcome the implementation gap and improve symptom control for cancer patients, the National Cancer Institute created the Cancer-Moonshot funded *I*mproving the *M*anagement of sym*P*toms during *A*nd following *C*ancer *T*reatment (IMPACT) Consortium.

**Methods:**

*S*ymptom Management *IM*plementation of *P*atient *R*eported Outcomes in *O*ncology (SIMPRO) is one of three research centers that make up the IMPACT Consortium. SIMPRO, a multi-disciplinary team of investigators from six US health systems, seeks to develop, test, and integrate an *e*lectronic *sy*mptom *m*anagement program (eSyM) for medical oncology and surgery patients into the Epic electronic health record (EHR) system and associated patient portal. eSyM supports real-time symptom tracking for patients, automated clinician alerts for severe symptoms, and specialized reports to facilitate population management. To rigorously evaluate its impact, eSyM is deployed through a pragmatic stepped wedge cluster-randomized trial. The primary study outcome is the occurrence of an emergency department treat-and-release event within 30 days of starting chemotherapy or being discharged following surgery. Secondary outcomes include hospitalization rates, chemotherapy use (time to initiation and duration of therapy), and patient quality of life and satisfaction. As a type II hybrid effectiveness-implementation study, facilitators and barriers to implementation are assessed throughout the project.

**Discussion:**

Creating and deploying eSyM requires collaboration between dozens of staff across diverse health systems, dedicated engagement of patient advocates, and robust support from Epic. This trial will evaluate eSyM in routine care settings across academic and community-based healthcare systems serving patients in rural and metropolitan locations. This trial’s pragmatic design will promote generalizable results about the uptake, acceptability, and impact of an EHR-integrated, ePRO-based symptom management program.

**Trial registration:**

ClinicalTrials.gov NCT03850912. Registered on February 22, 2019. Last updated on November 9, 2021.

## Administrative information

**Note:** the numbers in curly brackets in this protocol refer to SPIRIT checklist item numbers. The order of the items has been modified to group similar items (see https://www.equator-network.org/reporting-guidelines/spirit-2013-statement-defining-standard-protocol-items-for-clinical-trials/).

**Table:** 

Title {1}	Implementation of patient-reported outcomes for symptom management in oncology practice through the SIMPRO research consortium: a protocol for a pragmatic type II hybrid effectiveness-implementation multi-center cluster-randomized stepped wedge trial
Trial registration {2a and 2b}	ClinicalTrials.gov, NCT03850912. Registered on February 22, 2019. Last updated on November 9, 2021.
Protocol version {3}	Protocol version 7.0; January 12, 2022.
Funding {4}	This research is supported by the National Cancer Institute of the National Institutes of Health; 1UM1CA233080-01.
Author details {5a}	Dana-Farber Cancer Institute/Brigham and Women’s Hospital, Boston, MA (Hassett, Uno, Cronin) Maine Medical Center, Portland, ME (Bian) Lifespan Cancer Institute and Brown University, Providence, RI (Dizon) West Virginia University Cancer Center, Morgantown, WV (Hazard Jenkins) Baptist Medical Center, Memphis, TN (Osarogiagbon) Dartmouth Hitchcock Medical Center, Lebanon, NH (Wong) Memorial Sloan Kettering Cancer Center, New York, NY (Schrag)
Name and contact information for the trial sponsor {5b}	This research is supported by the National Cancer Institute of the National Institutes of Health; 1UM1CA233080-01.
Role of sponsor {5c}	The content of this protocol paper is solely the responsibility of the authors. It does not necessarily reflect the official views of the National Institutes of Health.

## Introduction

### Background and rationale {6a}

In the USA, nearly 1.9 million people will be diagnosed with cancer in 2022 [1]. In recent years, improved therapies have resulted in decreased mortality, but cancer-related morbidity remains substantial [2–6]. Deficits in symptom management, including sparse patient-clinician communication between clinic visits, contribute to considerable morbidity for cancer patients. Poor symptom control decreases quality of life, increases emergency care needs [7–9], and deters patients from receiving effective therapy [10, 11]. Healthcare in the USA is predominantly structured to be reactive, not proactive, with missed opportunities to optimize symptom control and ineffective strategies to anticipate, prevent and monitor adverse symptoms before they escalate [12].

Growth in internet access and proliferation of smartphones and tablets has created an opportunity to support improved cancer care delivery, particularly symptom control. In 2021, 93% of adults in the USA reported using the internet and 85% owned a smartphone [13, 14]. Even among the elderly and poor, web use is rising for many reasons including managing health needs [15, 16]. Mobile phones and web access create opportunities for patient-clinician communication to optimize symptom management beyond face-to-face encounters [17]. Patient engagement has been called the “blockbuster” drug [18, 19] of the twenty-first century as motivated patients demonstrate improved well-being and health outcomes [20, 21]. Strong theoretical foundations from social cognitive theories of self-efficacy [22, 23] and the chronic care model [24] support the importance of patient engagement [25–29] to minimize toxicities of cancer treatment.

Patient-reported outcomes are a patient’s direct report of their health, quality of life, or functional status without interpretation from the clinical care team. Symptom tracking and management via electronic patient-reported outcomes (ePROs) is one promising mechanism by which Internet access may be harnessed to reduce symptom burden among cancer patients through the use of patient portals. In research settings, utilization of ePROs has been shown to decrease symptom burden [30], improve quality of life, reduce acute care needs [31], and extend survival [32]. There are two primary mechanisms for these impacts. First, systematic collection of ePROs can support the recognition of problematic symptoms and may encourage self-management [33]. Second, systematic reporting of symptoms can trigger clinical interventions to improve symptom management outside of regularly scheduled clinic visits. Enhanced communication facilitated by technology may make the health care system more responsive to patient needs [34, 35].

Although prior ePRO work is promising [29, 32], major knowledge gaps persist regarding the optimal implementation of ePROs in routine oncologic care. First, the effectiveness of ePROs has primarily been studied in clinical trials at well-resourced academic cancer centers, leaving less known about their implementation in community settings [36, 37]. Second, existing ePRO platforms have not been integrated into the electronic health record (EHR), requiring patients and clinicians to access separate systems to view symptom reports, thereby introducing inefficiency and barriers to adoption [38]. Third, ePRO systems have rarely integrated effective coaching strategies for enhanced symptom management. Lastly, optimal implementation strategies for integrating ePROs into the standard clinical workflow are unknown.

To address these gaps and others identified by the Cancer Moonshot^SM^ [39], the National Cancer Institute funded the Improving the Management of symPtoms during and following Cancer Treatment (IMPACT) Consortium [40]. Its goals are to improve symptom control for cancer patients through systematic symptom reporting and guideline-based clinical management [41]. IMPACT is made up of three research centers, including the *S*ymptom Management *Im*plementation of *P*atient-*R*eported Outcomes in *O*ncology (SIMPRO) Consortium. SIMPRO is led by a multi-disciplinary team of investigators from six US-based health systems (Table 1) who are developing, testing, and implementing an EHR-integrated symptom management program in large and small, rural and metropolitan, academic and in community-based clinics for medical oncology and surgical patients.

**Table 1:**
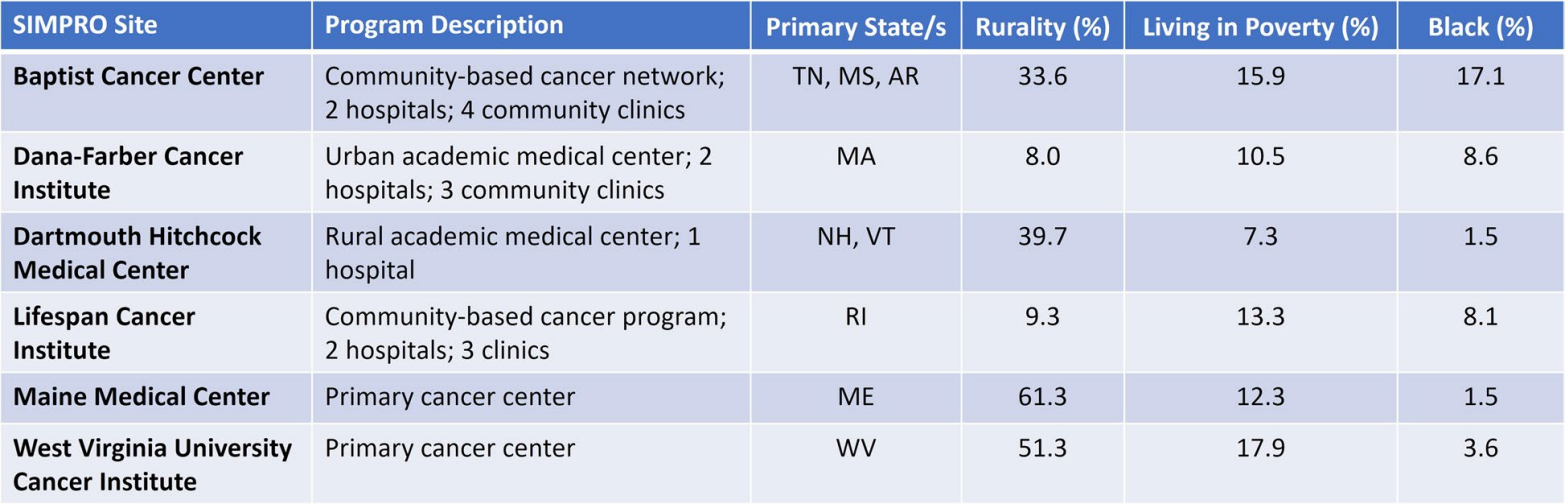
Characteristics of SIMPRO sites

### Objectives {7}

The research goals of the SIMPRO Consortium are:

1. Create and refine an ePRO-based symptom management system (eSyM) and integrate it into the EHR and routine clinical workflow
2. Determine the effectiveness of eSyM on health outcomes. Specifically, evaluate the impact on:
  ◦ Healthcare utilization, measured by the use of emergency and acute care
  ◦ Cancer care delivery, including duration and delay of medical treatments, and the need for re-operation
  ◦ Patient-centered outcomes, including self-efficacy and symptom burden
  ◦ Patient satisfaction with care

3. Evaluate the facilitators and barriers to implementation of eSyM from the patient, clinician, and organizational perspectives using an implementation science framework [42, 43]

A prior manuscript described the eSyM build and design process [44]. The current manuscript outlines the design used to evaluate the effectiveness of eSyM. Detailed methods for describing facilitators and barriers to implementation will be highlighted in a future manuscript.

### Trial design {8}

**Fig. 1:**
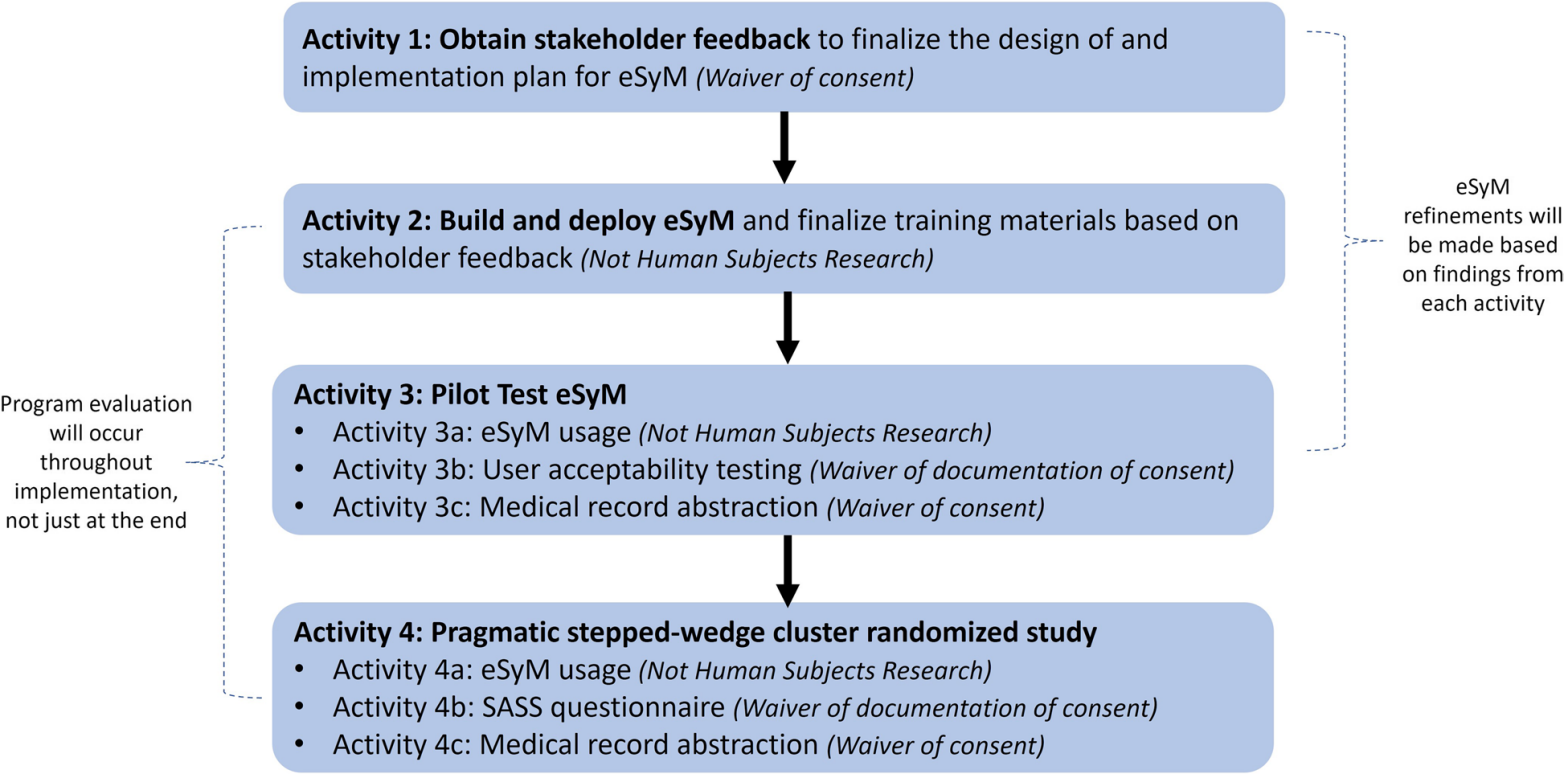
SIMPRO research consortium project schema

Overall, the SIMPRO project consists of four key activities (Fig. 1). The first three activities focused on building the infrastructure needed to support participating sites, configuring the eSyM program, and pilot testing the intervention. The fourth activity — described in this manuscript — is the conduct of a pragmatic type II hybrid effectiveness-implementation stepped wedge cluster-randomized trial to evaluate the effectiveness of eSyM. The Western Institutional Review Board (WIRB) approved the protocol as a minimal risk research study on November 25, 2018 (protocol #20182593), with a Health Insurance Portability and Accountability Act (HIPAA) waiver.

**Fig. 2:**
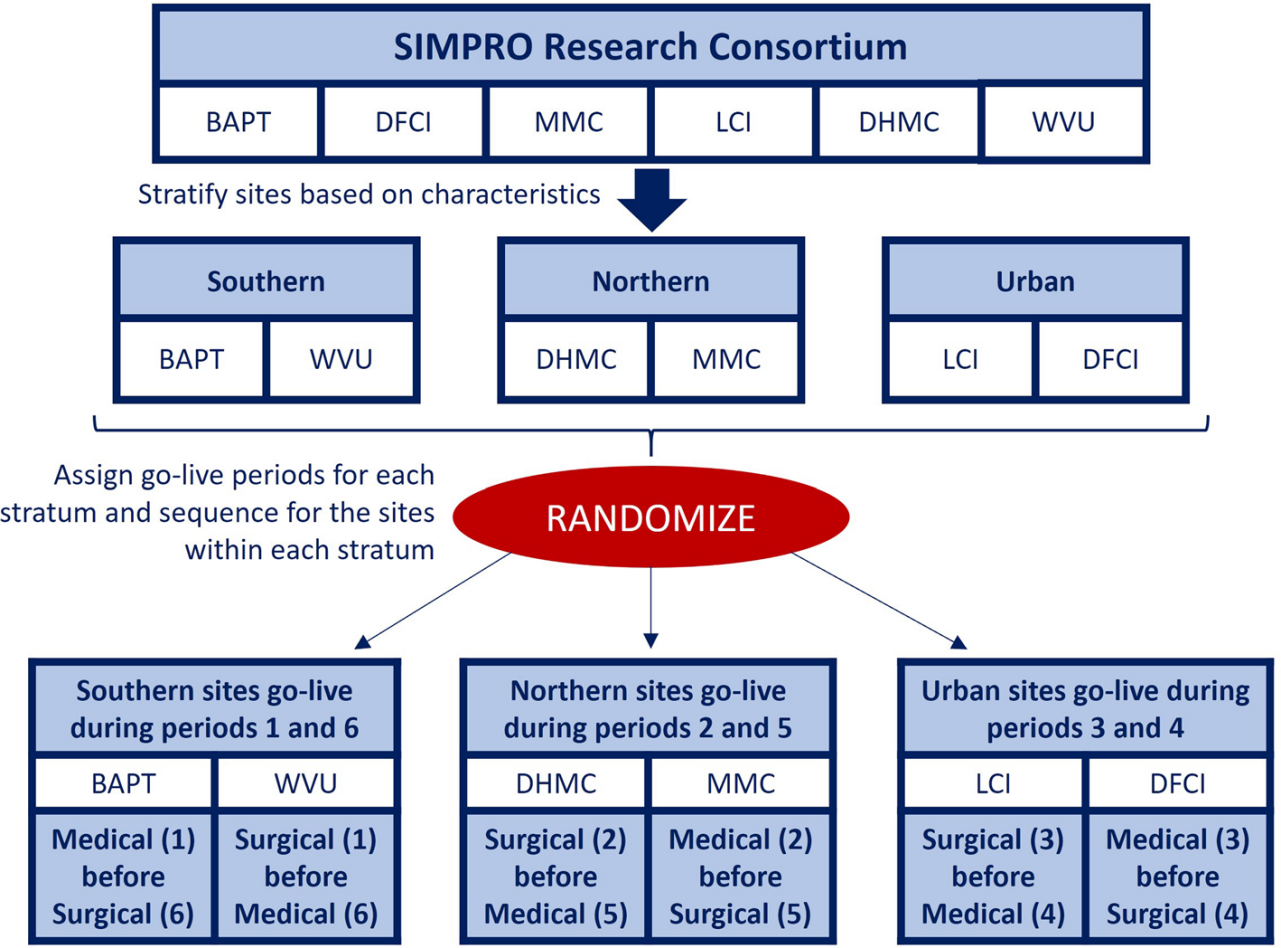
SIMPRO consortium randomization schema. Abbreviations: BAPT Baptist, WVU West Virginia University, MMC Maine Medical Center, DHMC Dartmouth Hitchcock Medical Center, LCI Lifespan Cancer Institute, DFCI Dana-Farber Cancer Institute, SIMPRO *S*ymptom Management *IM*plementation of *P*atient *R*eported *O*utcomes in Oncology

Each healthcare system (referred to as a site) is the unit of randomization. Within each site, two versions of eSyM are deployed — one for medical oncology patients and one for surgery patients. The randomization schema (Fig. 2) was constructed to ensure that (a) each site launches the two versions of eSyM during different time periods, (b) three sites launch eSyM-medical oncology first and the other three launch eSyM-surgery first, and (c) sites with similar characteristics (i.e., geographic location and metropolitan/rural setting) deploy eSyM on different schedules. The stepped wedge design includes seven time periods.

Consistent with the stepped wedge design [45], patients are involved in the trial in one of two ways:

1. Eligible patients seen at a participating site before eSyM deployment, and therefore not exposed to the intervention, form a “control group”
2. Eligible patients seen at a participating site after eSyM deployment form the “intervention group.” They are exposed to all aspects of the intervention, including eSyM questionnaires, reminders, symptom self-management tip sheets, and population management tools.

Outcomes are collected from control and intervention patients in the following ways:

1. For all patients, automated data extracts from the electronic medical record are used to ascertain demographics, treatments, healthcare service utilization, and outcomes.
2. A subset of eligible patients is randomly selected to complete a one-time research questionnaire asking about their *S*elf-efficacy, *A*ttainment of information needs, *S*ymptom burden, and *S*atisfaction with care (SASS). Among intervention patients, the SASS questionnaire includes additional questions about eSyM program engagement and use.

As a type II hybrid effectiveness-implementation study, facilitators and barriers to implementation are assessed throughout the project (this aspect of the project will be discussed in detail in a future manuscript).

## Methods: participants, interventions, and outcomes

### Study setting {9}

eSyM is deployed through the SIMPRO Consortium at six US-based healthcare institutions: Baptist Memorial Health Care, Memphis, TN; Dana-Farber Cancer Institute, Boston, MA; Dartmouth Hitchcock Medical Center, Lebanon, NH; Lifespan Health System, Providence, RI; Maine Medical Center, Portland ME; and West Virginia University, Morgantown, WV. These health systems represent a diverse mix of academic/community, rural/metropolitan, North-eastern and Southern community-based cancer centers (Table 1).

### Eligibility criteria {10}

Eligible patients are at least 18 years old. Medical oncology patients must be scheduled to receive a new chemotherapy treatment plan in the next 30 days; have a relevant ICD-10 diagnosis code for a thoracic, gastrointestinal, or gynecologic malignancy; and have an encounter at a participating medical oncology site. Surgical patients must be scheduled for surgery commonly used to treat a thoracic, gastrointestinal, or gynecologic malignancy; have a surgery date in the next 30 days; and have an encounter at a participating surgery site. Patients undergoing surgery are considered eligible regardless of whether they have a cancer diagnosis at the time of surgery, as pathologic confirmation of cancer may not be available until several weeks after surgery. We exclude incarcerated individuals as a protected population; pregnant women and cognitively impaired adults are eligible because of the negligible risk of the intervention. Although sites could expand eSyM to populations beyond the above-listed, only the specified patient population is included in research analyses.

A subset of eSyM-eligible patients is randomly selected to participate in the SASS sub-study and complete either a control or intervention SASS questionnaire (SASS-control or SASS-intervention, respectively). Selection of SASS questionnaire participants is stratified by cluster and period to ensure even spread. There are no additional eligibility or exclusion criteria for the SASS sub-study other than agreeing to complete a one-time English-language questionnaire.

### Who will take informed consent? {26a}

Since eSyM is implemented and tested for use in routine clinical practice and was deemed standard-of-care and minimal risk, the IRB waived the individual informed consent requirement for both eSyM participation and medical records review.

### Additional consent provisions for collection and use of participant data and biological specimens {26b}

Patients asked to complete a SASS questionnaire are informed of the sub-study’s details by research coordinators and receive a letter outlining the elements of informed consent. The requirement for formal documentation of written informed consent was waived by the IRB. Additionally, this trial does not involve collecting biological specimens for storage.

## Interventions

### Explanation for the choice of comparators {6b}

We will compare patients who started chemotherapy or had surgery before eSyM deployment (i.e., control episodes) with patients who started chemotherapy or had surgery after eSyM deployment (i.e., intervention episodes). This pre-post design allows each site to provide its own control group and likely reduces the potential for confounding on cluster- and possibly individual-level characteristics. Intervention patients are encouraged, but not required, to use eSyM. Thus, the intervention group is defined by exposure to, not use of, eSyM. Patients who receive multiple chemotherapy treatments or surgeries can contribute multiple episodes to the analysis. For example, a patient who received surgery during the control period and chemotherapy during the intervention period would contribute an episode to the surgery control cohort and a separate episode to the chemotherapy intervention cohort.

### Intervention description {11a}

The eSyM program [44] includes the following core features: (1) EHR registries that automatically identify patients for eSyM activation, (2) patient portal questionnaires that assess symptoms at regular intervals for a defined period after chemotherapy or surgery (the questionnaires are based on the validated Patient Reported Outcome version of the Common Terminology Criteria for Adverse Events [PRO-CTCAE®] plus two pictogram-based questions assessing overall well-being and performance status on a Likert scale), (3) patient self-management symptom tip sheets, (4) patient-facing views of past symptom reports, (5) care-team in-basket alerts for reported severe symptoms, (6) clinician-facing symptom reports and charting tools, and (7) population management reports [44].

Since teams caring for medical oncology and surgery patients may not overlap, and to accommodate differences in hospital system structures and priorities, eSyM was designed with the flexibility to be deployed for both medical oncology and surgery patients, exclusively or in combination. Deploying the medical oncology and surgery versions of eSyM at different time points helps account for co-existing trends that are unrelated to the intervention. The features of eSyM medical oncology and surgery are nearly identical. A patient can only be active on one version of eSyM at a time but can be active on different versions of eSyM sequentially. To use eSyM, patients must either have access to the patient portal (which requires having a smartphone or computer that has internet access) or be provided a tablet during clinic visits (available at two sites).

### Criteria for discontinuing or modifying allocated interventions {11b}

eSyM automatically becomes available to eligible patients via the patient portal 1 day after either chemotherapy starts or hospital discharge after surgery. The program automatically discontinues after 180 days for medical oncology patients and after 60 days for surgical patients. Patients who do not wish to participate can either ignore questionnaire reminders or ask their care team to discontinue them.

### Strategies to improve adherence to interventions {11c}

**Fig. 3:**
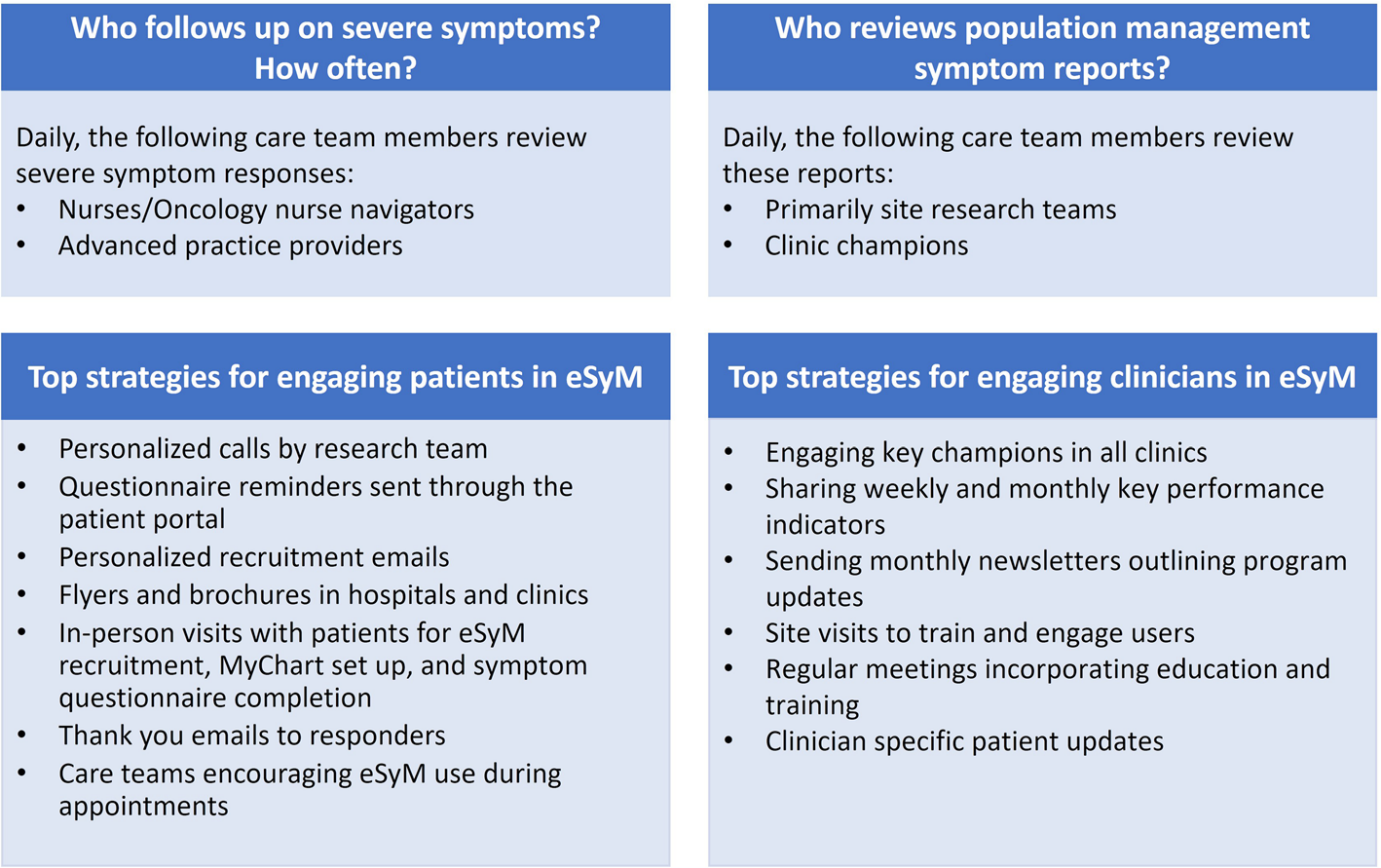
Description of processes and implementation strategies at SIMPRO sites

We use several implementation science strategies to support stakeholder (i.e., patient and clinician) engagement. Educational materials and outreach efforts foster patient use of the program, and training materials and outreach efforts increase care team engagement with the program. Sites routinely employ a coordinator to monitor eSyM patients. Coordinators approach patients to encourage eSyM engagement and assist with program setup, questionnaire submission, technical questions, and care team training. In addition to completing eSyM symptom questions via the patient portal, some sites enable symptom reporting via an internet tablet in clinic. For patients unable to complete questionnaires independently, proxy reporting by a family member or close contact is encouraged. Figure 3 outlines specific implementation strategies recorded to date. Throughout the project, additional implementation strategies are being documented by the site project coordinators in a central database.

### Relevant concomitant care permitted or prohibited during the trial {11d}

eSyM is deployed as a standard-of-care intervention. It neither requires nor precludes concomitant care during the trial.

### Provisions for post-trial care {30}

The trial makes no provisions for post-trial care. After the trial, sites may choose to continue using eSyM with or without modifications, or they may discontinue its use.

### Outcomes {12}

The primary outcome is the occurrence of an emergency department treat-and-release (EDTR) event within 30-days of chemotherapy start or surgical discharge (binary outcome). Secondary clinical outcomes for surgery patients include time to initiation of adjuvant chemotherapy, time to re-operation, time to re-admission, and overall survival. Secondary outcomes for medical oncology patients include time to first chemotherapy treatment, time to delay or dose modification, time to chemotherapy discontinuation, time to admission, and vital status. Patients are followed for 1 year after the treatment start date. Secondary outcomes focus on events through 90, 180 and 360 days. For the SASS sub-study, secondary patient-reported outcomes are evaluated approximately 30–60 days after the treatment initiation date.

### Participant timeline {13}

eSyM becomes available to medical oncology patients the day after the first treatment for a new chemotherapy plan is completed (i.e., cycle 1 day 2). Medical oncology patients can respond to eSyM’s PRO-CTCAE-based symptom questionnaires up to twice per week for 180 days. eSyM becomes available to surgery patients the day after hospital discharge following an eligible surgical procedure. Surgery patients can respond to questionnaires on a tapering schedule, up to 3 times per week for weeks 1–2, up to twice per week for weeks 3–4, and up to once a week for weeks 5–8. If a patient transitions from medical oncology to surgery, or vice versa, then the medical oncology version of eSyM discontinues and the surgery version becomes active. Therefore, only one program is active at a time. There is no limit on the number of times patients may restart the eSyM program for new qualifying treatment plans or surgeries.

### Sample size {14}

We calculated the sample size to have 80% power to detect a difference in the primary outcome of EDTR between the control and invention groups using the stepped wedge clustered randomized design [46]. Thirty-day EDTR rates are estimated to vary between 8 and 15% for the control group. We hypothesized that EDTR rates will be 3–4% lower in the eSyM-intervention group. We based control group rates on EDTR rates derived from HCUP data, institutional data, and early phase analyses from CMMI’s Oncology Care Model (OCM) for Baptist Memorial, the only OCM participant among our six sites. Table 2 shows the required sample size to have 80% power to detect a difference between groups, at two-sided alpha level 0.05, using the stepped wedge cluster-randomized design [46].

**Table 2:**
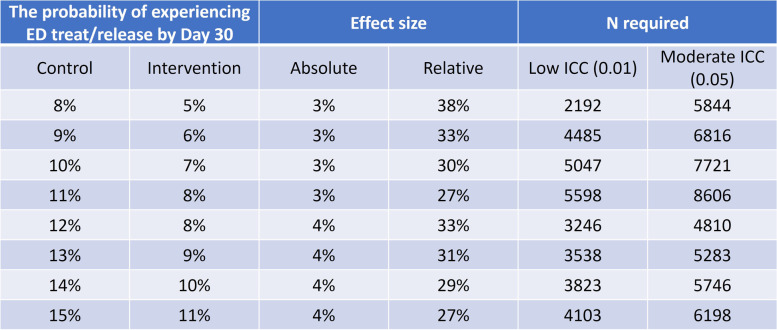
Required total sample size with various scenarios for 80% power

Analyses of inter-institutional variation in hospitalization and ED visit rates from AHRQ’s statewide databases indicate low intraclass correlations (ICC) [47, 48]. A low ICC (0.01) is expected for this study because (1) the intervention is deployed with the same technology across hospitals and (2) adjustments are made for variation in baseline risk via generalized linear mixed models such that potential differences in case-mix among hospitals will be negligible. Given these factors, a conservative estimate would suggest that 6048 participants will provide adequate power to evaluate the primary outcome. This equates to a minimum of 504 medical oncology and 504 surgical patients at each of the 6 participating sites. The protocol does not mandate equal distribution of participants among participating sites. Since eSyM is being deployed as a broad-based system-level implementation, it is anticipated that the trial will have a much higher N, allowing for review and analysis of subgroups of interest.

### Recruitment {15}

eSyM-eligible patients are identified automatically using two Epic-based patient registries — one for medical oncology and one for surgery. Thus, patients are not actively recruited. After patients are added to a registry, the system automatically assigns the symptom-based questionnaire series. Patients must have an active patient portal account to use most eSyM features. To increase awareness and engagement, patients receive a welcome message and instructions via their patient portal. Participants may also receive additional program information via pamphlets, telephone calls, emails, and/or personal communications during pre-operative or chemotherapy appointments. During these interactions, study team members assist with patient portal set-up (if needed), eSyM program education, and first questionnaire completion.

## Assignment of interventions: allocation

### Sequence generation {16a}

**Fig. 4:**
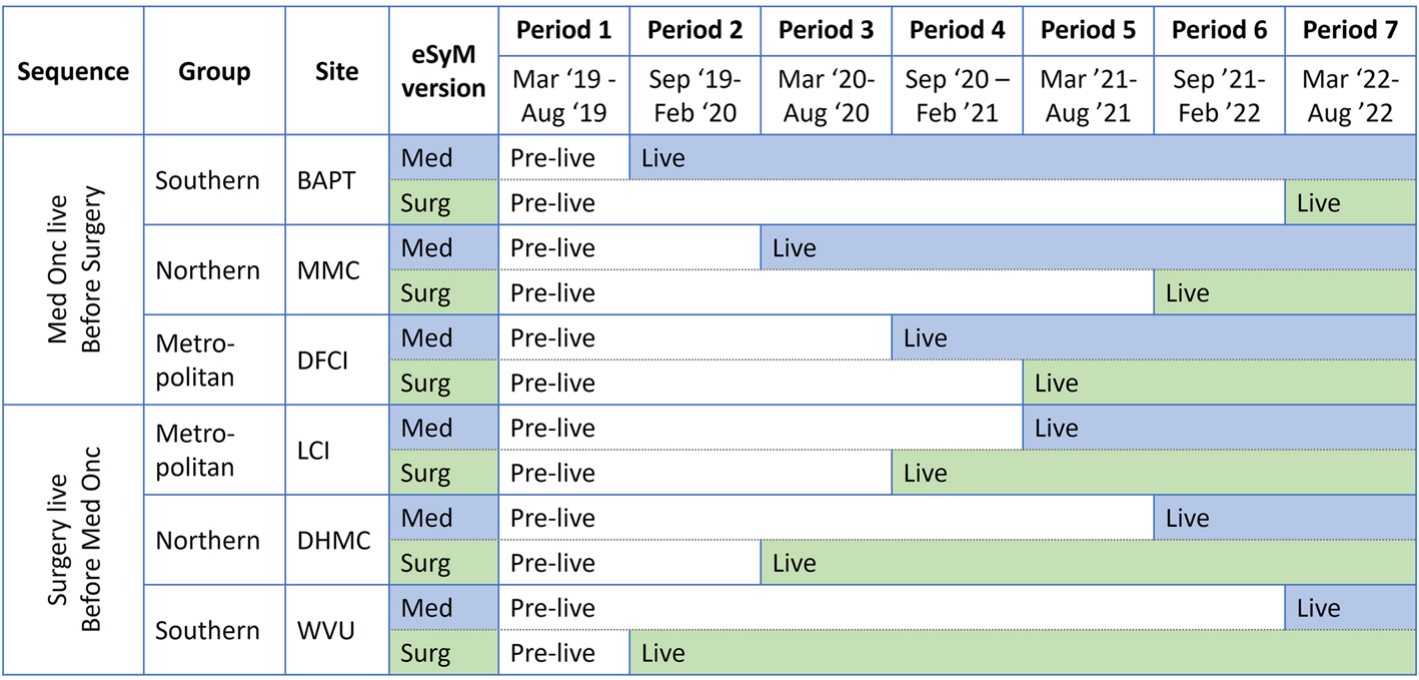
SIMPRO stepped wedge randomization schema. Abbreviations: BAPT Baptist, WVU West Virginia University, MMC Maine Medical Center, DHMC Dartmouth Hitchcock Medical Center, LCI Lifespan Cancer Institute, DFCI Dana-Farber Cancer Institute, SIMPRO *S*ymptom Management *IM*plementation of *P*atient *R*eported *O*utcomes in Oncology

To determine the site activation sequence, sites were first categorized into three paired groups: Southern/non-metropolitan (WVU and Baptist), Northern/non-metropolitan (Maine and Dartmouth), or Northern/metropolitan (Dana-Farber and Lifespan). The intention was to ensure that sites with similar characteristics would have different activation sequences. Then, site pairs were randomly assigned to time periods: Southern deploys during the 1st and 6th periods, Northern deploys during the 2nd and 5th periods, and Metropolitan deploys during the 3rd and 4th periods. Finally, within each group, one site was randomly assigned to deploy medical oncology before surgery and the other to deploy surgery before medical oncology (Fig. 4). With six participating sites, each deploying eSyM on two separate occasions, there are twelve total deployments (“go-lives”). Initiations were staggered, so that (a) each period includes one medical oncology and one surgery deployment, (b) each site deploys eSyM-medical oncology and eSyM-surgery during different time periods, and (c) each site has the same total time exposed to eSyM.

### Concealment mechanism {16b}

The randomization sequence was not concealed as sites needed to plan for their eSyM deployments with their technical, operational, and clinical teams.

### Implementation {16c}

The allocation sequence was generated randomly by the study statistician at the project coordinating center on November 6, 2018.

## Assignment of interventions: blinding

### Who will be blinded {17a}

There is no blinding for patients or sites. eSyM is deployed as a standard-of-care intervention by health systems, such that all patients within a health system have the same intervention exposure, reducing the need for blinding. Post-deployment, all patients are notified of the eSyM program and can choose to participate or not, and all site staff are educated about eSyM to foster successful implementation.

### Procedure for unblinding if needed {17b}

This stepped wedge trial does not require procedures for unblinding because the eSyM intervention is being administered through standard-of-care clinical practice with no blinding for patients or sites. The study design is open label so blinding will not occur.

## Data collection and management

### Plans for assessment and collection of outcomes {18a}

Outcomes for control and intervention patients are collected via data extracts from the Epic EHR using custom structured query language (SQL) queries run by each site’s data warehouse team. The primary intervention outcomes include encounters (i.e., ambulatory visits, ED visits, and admissions); chemotherapy treatments, durations, and discontinuations; procedures; and patient portal and eSyM utilization. For the SASS sub-study, patient-reported self-efficacy, symptom burden, and satisfaction with care are assessed through a one-time questionnaire approximately 30–60 days after chemotherapy or surgery. A minimum of 1800 questionnaires are collected across sites, of which 900 are collected before and 900 after eSyM deploys. The SASS questionnaire varies modestly based on the type of treatment (medical oncology vs. surgery), the timing relative to eSyM deployment (before vs. after), and patient use of eSyM’s symptom monitoring questionnaires (responder vs. non-responder).

### Plans to promote participant retention and complete follow-up {18b}

For eSyM patients, an introductory message explaining the program is sent via the patient portal, and automated reminders are sent at pre-defined intervals (approximately twice per week) encouraging symptom questionnaire completion. At some sites, eSyM patients receive additional reminders to engage with the program through phone calls, emails, or in-clinic touch points. The SIMPRO coordinating center regularly updates each site with key performance indicators, including eSyM participation rates, to encourage sites to increase their patients’ use of eSyM. Patients who choose to stop receiving care at a SIMPRO site will have an incomplete follow-up as their encounters will no longer be captured by the site’s EHR system. For patients taking part in the SASS questionnaire, there is no specific retention or follow-up plan.

### Data management {19}

**Fig. 5:**
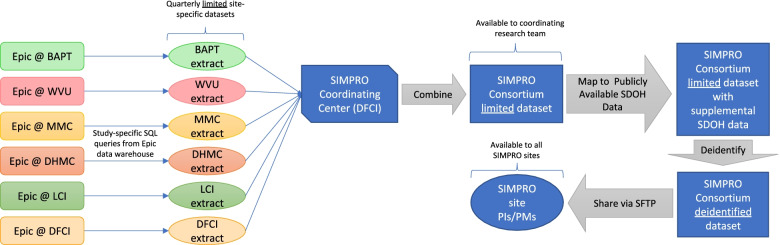
SIMPRO Consortium data collection and transfer plan. Abbreviations: BAPT Baptist, WVU West Virginia University, MMC Maine Medical Center, DHMC Dartmouth Hitchcock Medical Center, LCI Lifespan Cancer Institute, DFCI Dana-Farber Cancer Institute, SIMPRO *S*ymptom Management *IM*plementation of *P*atient *R*eported *O*utcomes in Oncology, SDOH social determinants of health, PI principal investigator, PM project manager

Patient data are collected via Epic and REDCap. Epic, a HIPAA-compliant EHR, is used to deploy eSyM and to capture demographic data, healthcare and patient portal utilization information, and clinical outcomes. REDCap, a HIPAA-compliant and 21 CFR Part 11 application, is used to collect responses to the SASS questionnaire, which asks about patient health information as well as perceptions and behaviors regarding patient portal and ePRO systems. Both systems employ user privileges, password protection and authentication, auto-logout settings, and audit trails. Study-specific procedures to maximize data security include controlled access, use of unique study identification numbers, extensive training, and regular quality checks. Once collected, data are deidentified and shared with the SIMPRO coordinating center via secure file transfer protocols in compliance with a fully executed multi-center data transfer agreement. Data collection and transfer procedures are outlined in Fig. 5.

### Confidentiality {27}

The study team has taken many steps to protect patient privacy and confidentiality, including training all staff in best practices and regulations, collecting research data using unique identification numbers, and using data collection systems that meet NIH data security standards. In addition, only limited deidentified data are transferred to the coordinating center.

### Plans for collection, laboratory evaluation, and storage of biological specimens for genetic or molecular analysis in this trial/future use {33}

This trial does not include collection, laboratory evaluation, or storage of biological specimens for genetic or molecular analysis.

## Statistical methods

### Statistical methods for primary and secondary outcomes {20a}

This type II effectiveness-implementation hybrid study will assess the impact of eSyM and describe the facilitators and barriers to eSyM having an impact [49]. The primary analysis will be conducted with the medical oncology and surgical cohorts combined. Descriptive statistics will be used to elucidate characteristics of clusters and participants; multilevel generalized linear regression models will be used to characterize relationships with outcomes controlling for other factors. For each outcome, modification of the intervention effect by cohort (i.e., surgery and medical oncology) will be assessed by including interaction tests. If the observed *p*-value for the test for interaction is greater than 0.2, the interaction term will be removed from the model and a common intervention effect will be estimated. If the observed *p*-value is below 0.2, analyses for the medical and surgical cohorts will be conducted separately and intervention effects will be estimated for each cohort.

The primary study outcome is the occurrence of an EDTR event within 30 days of chemotherapy start or surgical discharge. This will be classified as a binary outcome: any or no EDTR within 30 days. Events occurring on the first day of chemotherapy infusion or the day of hospital discharge will be excluded, because they could not have been impacted by the eSyM program. Because each patient could experience multiple episodes during the study period (i.e., start more than one chemotherapy region or have more than one surgery), the analysis could include multiple data points from the same patient. Multilevel generalized linear regression with the logit-link will account for within-subject correlations and confounding by known covariates (e.g., age, sex, race/ethnicity, employment status, marital status, socio-economic status, type of cancer, type of surgery or chemotherapy, treating facility, number of co-morbidities). Factors included in the generalized linear regression models will include intervention, time, hospital (fixed effect), subject (random effect), and other potentially relevant predictive factors. The models will include random intercepts; we may explore inclusion of random slopes to assess for temporal changes. If a mixed effect model does not converge, we will use a generalized estimating equation approach instead. Results will be reported as odds ratios with 95% confidence intervals.

Key secondary outcomes include the initiation of chemotherapy for surgery patients and the duration of chemotherapy for medical oncology patients. For surgery patients, we will look at the time to initiation of chemotherapy and whether chemotherapy is given within 90 days of discharge. For medical oncology patients, we will assess the time to discontinuation of chemotherapy and the total duration of chemotherapy treatment. For all patients, we will assess response to eSyM questionnaires and utilization of healthcare services. Multilevel generalized linear regression models will be used with an appropriate link function (e.g., logit-link for binary outcomes, log-link for count data, and time-to-event outcomes [e.g., time to admission/re-admission, time to surgery, time to death], and identity-link for continuous outcomes [e.g., self-reported quality of life]), to compare intervention vs. control patients while also controlling for other potential confounding variables.

Participants in the SASS sub-study self-report five PROMIS short-form domains: pain, fatigue, depression, anxiety, and physical function. Past studies have reported minimally important difference ranges for these domains, which are approximately 3–5 using a *T*-score scale [50] or 0.5 using a standard deviation scale [51–53]. To systematically assess patients’ care experience, participants in the SASS sub-study self-report using the Consumer Assessment of Healthcare Providers and Systems (CAHPS) cancer care surveys for drug therapy and surgery [54, 55]. For each of the five PROMIS domains and two CAPHS surveys, we will compare scores for eSyM-intervention versus control patients, adjusting for case-mix and other potential confounding variables.

### Interim analyses {21b}

eSyM utilization is assessed monthly to guide and adapt implementation efforts using two patient-level metrics: (1) response to at least one symptom monitoring questionnaire and (2) response to multiple symptom monitoring questionnaires. We had not planned a formal interim analysis, but the COVID-19 pandemic forced a change in plans. Although eSyM deployments have occurred on schedule, the COVID-19 pandemic has impacted the eSyM project in many ways. Most notably, emergency department visits plummeted during the pandemic [56]. Since the project’s primary outcome is 30-day EDTR, the actual proportion of the event occurrence could be lower than estimated, adversely impacting study power. Therefore, we plan to conduct an interim analysis to estimate the event rate for the intervention cohort, without comparing the intervention and control groups. If needed, we will amend the protocol to extend accrual.

### Methods for additional analyses (e.g., subgroup analyses) {20b}

Analyses to assess for heterogeneity in treatment effects are considered exploratory and will be limited to pre-specified salient domains. Subgroups of interest are (1) computer experience, (2) rurality, (3) age (less than 70 versus greater than or equal to 70), (4) cancer or surgery type, and (5) patient versus patient-proxy reporting. We will also look to see if the intervention has a differential impact over time and by cluster.

### Methods in analysis to handle protocol non-adherence and any statistical methods to handle missing data {20c}

Patients exposed to protocol non-adherence will be excluded from the analysis. To evaluate the robustness of the results for the primary analysis, sensitivity analyses will be performed with various approaches to handle missing observations, including best or worst case imputations, mean value imputation, and multiple imputations [57]. For EHR data, substantial missing data is not anticipated but, if needed, multiple imputations will be performed and results integrated using Rubin’s method [57]. For SASS questionnaire patient response data, we will use several methods to handle missing observations, including mean value, worst-case, best-case, and multiple imputations [57].

### Plans to give access to the full protocol, participant-level data, and statistical code {31c}

All data are stored in a master deidentified dataset at the SIMPRO Consortium coordinating center. In accordance with IMPACT funding requirements and the SIMPRO data use agreement, a deidentified data set will be shared with the National Institutes of Health (NIH) and collaborators. Investigators can request the data collected from this study for new research. Requests must be approved by WIRB, the Dana-Farber IRB, and the NIH prior to sharing.

## Oversight and monitoring

### Composition of the coordinating center and trial steering committee {5d}

eSyM is deployed through the multi-center SIMPRO Consortium. The coordinating center (Dana-Farber) oversees administrative grant management, Epic software configuration, data analysis, and overall project and consortium coordination. The coordinating center includes a co-principal investigator, two statisticians, a program manager, and two research coordinators. Members of the coordinating center, along with the principal investigators from each participating SIMPRO site and the eSyM technical lead, provide oversight and monitoring of data collection and management.

### Composition of the data monitoring committee, its role, and reporting structure {21a}

This is a minimal risk study assessing the impact of a standard-of-care implementation; there is no data monitoring committee.

### Adverse event reporting and harms {22}

This is a minimal risk, standard-of-care implementation; routine adverse event reporting is not conducted.

### Frequency and plans for auditing trial conduct {23}

The Epic extracts are audited quarterly by each site and by the SIMPRO coordinating center. Patient medical records are only accessible at individual sites; they can only be audited at the request of a site’s principal investigator. All other study records can be audited at the request of the project and site principal investigators. Audits will not be conducted by external groups.

### Plans for communicating important protocol amendments to relevant parties (e.g., trial participants, ethical committees) {25}

All protocol amendments are prepared, approved, and submitted to WIRB by the SIMPRO coordinating center. Once approved, memos are circulated to site project managers for record-keeping and submission to local IRBs in compliance with local policies. As this is a minimal risk study, patients are not notified of protocol amendments.

### Dissemination plans {31a}

The SIMPRO Consortium plans to share results with participants, healthcare professionals, institutions, and software developers. At the conclusion of the study, all participants will be directed to clinicaltrials.gov and PubMed to view results. For healthcare professionals, results will be made available through manuscript publications and conference presentations. The study team is working with Epic to make eSyM available to all Epic-based healthcare intuitions via Epic’s User Web, SharePoint, and annual meetings. The SIMPRO website (www.esymcancermoonshot.org) includes build information, symptom management tip sheets, workbooks, and other resources.

## Discussion

The SIMPRO consortium is conducting a pragmatic type II hybrid effectiveness-implementation stepped wedge cluster-randomized trial to assess the impact of a symptom management program for patients treated with cancer chemotherapy or surgery in the routine care setting. To date, key successes include (1) creating an ePRO-based symptom management program that is integrated into a widely used EHR system; (2) deploying eSyM across six health systems; (3) adapting eSyM, and its associated implementation strategies, based on feedback from patients and clinicians; (4) providing access to symptom monitoring and management tools to thousands of cancer patients; and (5) beginning to collect data from six versions of the Epic EHR using a common data model. The pragmatic design employed by this protocol will enable systematic analysis of the effectiveness of a novel care delivery intervention in a real-world setting.

This trial has faced several challenges that have underscored the importance of the adaptive approach:

*Operational*: As a dynamic implementation project, eSyM is continuously refined. Differing clinic structures, technical capabilities, and leadership engagement across the sites impact the consistency, feasibility, and acceptability of the program. Program needs change frequently based on real-time feedback from patients and clinicians. Intervention and protocol modifications are considered on an ongoing basis. Optimizing the program is labor-intensive and operationally challenging.

*Technical*: Partnering with Epic to develop eSyM has presented advantages (e.g., a robust EHR with a secure HIPAA-compliant patient portal), but technical challenges have nevertheless arisen. Healthcare institutions have unique EHR configurations and site-specific policies that can add time and complexity to system configuration and deployment efforts. Epic functional capabilities, such as limited user-interface display options, messaging capabilities, and notification settings, constrain what can be built into the system.

*Administrative*: Deploying eSyM at six separate institutions has required substantial coordination. Each site has unique staffing, processes, and procedures. Multi-center IRB submissions, EHR builds, data use agreements, data extraction procedures, and funding agreements are needed to support this project, which requires substantial administrative work by each site and the coordinating center.

*Clinician*: A key and persistent challenge has been clinician buy-in to a new symptom management program. eSyM is designed to integrate into existing workflows, but it still introduces new processes that have generated stress amidst pandemic-related work pressures and staffing shortages. We have adapted eSyM in response to clinician feedback. For example, the original questionnaire asked patients to report symptoms experienced over the last 7 days, but nurses expressed concern that this led patients to report symptoms that have resolved. Since the primary goal of eSyM is early identification and treatment of symptoms to prevent escalation, we revised the recall period to ask about symptoms experienced in the preceding 24 h. Effects of this modification will be analyzed.

*Patient*: Some patients engage with eSyM’s questionnaires and educational resources more than others. Barriers to engagement include limited access to technology (e.g., computers, smartphones, internet access), limited understanding of eSyM program benefits, and skepticism that healthcare providers will use eSyM reports to guide management decisions. Site teams review this feedback routinely and look for strategies that encourage regular use of the program.

Although the COVID pandemic has created many challenges, not all its impacts have been detrimental. It has prioritized telehealth in an unprecedented way, and it has provided a strong external impetus to engage stakeholders, including healthcare administrators, clinicians, and patients/caregivers, on the use of novel technologies and the need for novel approaches to healthcare delivery. A key aspect of our study design involves a detailed analysis of implementation outcomes, including patient adoption, clinician utilization, ePRO sustainability, penetration and scalability for symptom management, and extent of ePRO systems adaptation over the course of the implementation. Qualitative and quantitative implementation outcomes data, which are collected from stakeholders throughout the project, will help identify facilitators and barriers to implementation across multiple levels.

## Trial status

The study protocol was originally approved as a minimal risk research study on November 25, 2018 (protocol #20182593). Current protocol version 6.0 was approved on June 23, 2021. Pre-intervention SASS questionnaire data collection began July 25, 2019. The first cohort of eSyM patient recruitment began on September 10, 2019. Patient recruitment is expected to conclude on August 31, 2022. Data collection is expected to conclude in the last quarter of 2023.

## Data Availability

All data collected during this study will be stored and used for future research. Any personal identifiers will be removed so that the information cannot be linked back to any patients. Per the SIMPRO data use agreement, a fully deidentified data set will be shared with the National Institutes of Health and IMPACT consortium member sites in accordance with funding requirements. Once data collection is complete (estimated 4th quarter of 2023), investigators can request the deidentified data collected from this study for new research. Requests must be sent to the study chairs (Drs. Deborah Schrag, Michael Hassett, Sandra Wong, and Raymond Osarogiagbon) and must be approved by the IRB as well as the NIH prior to sharing. All relevant materials and study results will be posted to clinicaltrials.gov at the conclusion of this study.

## Abbreviations

EHR: Electronic health record
SASS: Self-efficacy, attainment of information needs, symptom burden, and satisfaction
eSyM: Electronic symptom management
SIMPRO: *S*ymptom Management *IM*plementation of *P*atient *R*eported *O*utcomes in Oncology
DFCI: Dana-Farber Cancer Institute
PRO: Patient-reported outcome
ePRO: Electronic patient-reported outcome
IMPACT: *I*mproving the *M*anagement of sym*P*toms during *A*nd following *C*ancer *T*reatment

## References

[1] Siegel RL, Miller KD, Fuchs HE, Jemal A (2022). Cancer statistics, 2022. CA Cancer J Clin.

[2] Cancer Statistics: National Cancer Institute; 2017 [Available from: https://www.cancer.gov/about-cancer/understanding/statistics.

[3] Cleeland CS (2007). Symptom burden: multiple symptoms and their impact as patient-reported outcomes. J Natl Cancer Inst Monogr.

[4] Hofman M, Ryan JL, Figueroa-Moseley CD, Jean-Pierre P, Morrow GR (2007). Cancer-related fatigue: the scale of the problem. Oncologist..

[5] Teunissen SC, Wesker W, Kruitwagen C, de Haes HC, Voest EE, de Graeff A (2007). Symptom prevalence in patients with incurable cancer: a systematic review. J Pain Symptom Manag.

[6] Temel JS, Pirl WF, Lynch TJ (2006). Comprehensive symptom management in patients with advanced-stage non-small-cell lung cancer. Clin Lung Cancer.

[7] Mayer DK, Travers D, Wyss A, Leak A, Waller A (2011). Why do patients with cancer visit emergency departments? Results of a 2008 population study in North Carolina. J Clin Oncol.

[8] Brooks GA, Abrams TA, Meyerhardt JA, Enzinger PC, Sommer K, Dalby CK (2014). Identification of potentially avoidable hospitalizations in patients with GI cancer. J Clin Oncol.

[9] Barbera L, Taylor C, Dudgeon D (2010). Why do patients with cancer visit the emergency department near the end of life?. Cmaj..

[10] Berry DL, Blonquist TM, Hong F, Halpenny B, Partridge AH (2015). Self-reported adherence to oral cancer therapy: relationships with symptom distress, depression, and personal characteristics. Patient Prefer Adherence.

[11] Fish JA, Prichard I, Ettridge K, Grunfeld EA, Wilson C (2015). Psychosocial factors that influence men's help-seeking for cancer symptoms: a systematic synthesis of mixed methods research. Psychooncology..

[12] Cohen E, Botti M (2015). Cancer patients’ perceptions of the barriers and facilitators to patient participation in symptom management during an episode of admission. Cancer Nurs.

[13] Pew Research Center. Internet/Broadband Fact Sheet 2021 [Available from: https://www.pewresearch.org/internet/fact-sheet/internet-broadband/.

[14] Pew Research Center. Mobile Fact Sheet 2021 [Available from: https://www.pewresearch.org/internet/fact-sheet/mobile/.

[15] Birkhoff SD, Smeltzer SC (2017). Perceptions of smartphone user-centered mobile health tracking apps across various chronic illness populations: an integrative review. J Nurs Scholarsh.

[16] Carroll JK, Moorhead A, Bond R, LeBlanc WG, Petrella RJ, Fiscella K (2017). Who uses mobile phone health apps and does use matter? A secondary data analytics approach. J Med Internet Res.

[17] Ernsting C, Dombrowski SU, Oedekoven M, Sullivan JLO, Kanzler M, Kuhlmey A (2017). Using smartphones and health apps to change and manage health behaviors: a population-based survey. J Med Internet Res.

[18] Koh HK, Brach C, Harris LM, Parchman ML (2013). A proposed ‘health literate care model’ would constitute a systems approach to improving patients’ engagement in care. Health affairs (Project Hope).

[19] Carman KL, Dardess P, Maurer M, Sofaer S, Adams K, Bechtel C (2013). Patient and family engagement: a framework for understanding the elements and developing interventions and policies. Health affairs (Project Hope).

[20] Hibbard JH, Mahoney E (2010). Toward a theory of patient and consumer activation. Patient Educ Couns.

[21] Coulter A, Ellins J (2007). Effectiveness of strategies for informing, educating, and involving patients. BMJ (Clinical research ed).

[22] Bandura A (1982). Self-efficacy mechanism in human agency. Am Psychol Assoc.

[23] Strecher VJ, DeVellis BM, Becker MH, Rosenstock IM (1986). The role of self-efficacy in achieving health behavior change. Health Educ Q.

[24] Coleman K, Austin BT, Brach C, Wagner EH (2009). Evidence on the chronic care model in the new millennium. Health affairs (Project Hope).

[25] Survey Snapshot: Health care providers on the problems of patient engagement design: NEJM catalyst; 2017 [Available from: https://catalyst.nejm.org/problems-patient-engagement-design/.

[26] Manary MP, Boulding W, Staelin R, Glickman SW (2013). The patient experience and health outcomes. N Engl J Med.

[27] McCabe C, McCann M, Brady AM (2017). Computer and mobile technology interventions for self-management in chronic obstructive pulmonary disease. Cochrane Database Syst Rev.

[28] Posadzki P, Mastellos N, Ryan R, Gunn LH, Felix LM, Pappas Y (2016). Automated telephone communication systems for preventive healthcare and management of long-term conditions. Cochrane Database Syst Rev.

[29] Basch E, Deal AM, Kris MG, Scher HI, Hudis CA, Sabbatini P (2016). Symptom monitoring with patient-reported outcomes during routine cancer treatment: a randomized controlled trial. J Clin Oncol.

[30] Snyder CF, Blackford AL, Aaronson NK, Detmar SB, Carducci MA, Brundage MD (2011). Can patient-reported outcome measures identify cancer patients’ most bothersome issues?. J Clin Oncol.

[31] Abelson JS, Symer M, Peters A, Charlson M, Yeo H (2017). Mobile health apps and recovery after surgery: what are patients willing to do?. Am J Surg.

[32] Basch E, Deal AM, Dueck AC, Scher HI, Kris MG, Hudis C (2017). Overall survival results of a trial assessing patient-reported outcomes for symptom monitoring during routine cancer treatment. Jama..

[33] McCorkle R, Ercolano E, Lazenby M, Schulman-Green D, Schilling LS, Lorig K (2011). Self-management: enabling and empowering patients living with cancer as a chronic illness. CA Cancer J Clin.

[34] Fisch MJ, Chung AE, Accordino MK (2016). Using technology to improve cancer care: social media, wearables, and electronic health records. Am Soc Clin Oncol Educ Book.

[35] Mobasheri MH, Johnston M, Syed UM, King D, Darzi A (2015). The uses of smartphones and tablet devices in surgery: a systematic review of the literature. Surgery..

[36] Basch E, Snyder C, McNiff K, Brown R, Maddux S, Smith ML (2014). Patient-reported outcome performance measures in oncology. J Oncol Pract.

[37] Snyder CF (2014). Using patient-reported outcomes in clinical practice: a promising approach?. J Clin Oncol.

[38] Roess A (2017). The promise, growth, and reality of mobile health - another data-free zone. N Engl J Med.

[39] National Cancer Institute. Cancer moonshot: NIH; 2022 [updated 2/2022. Available from: https://www.cancer.gov/research/key-initiatives/moonshot-cancer-initiative.

[40] National Cancer Institute. Improving the Management of symPtoms during And following Cancer Treatment (IMPACT) [Available from: https://healthcaredelivery.cancer.gov/impact/.

[41] Hassett MJ, Schrag D, Osarogiagbon R, Wong S, Kroner B, Cella D, Cheville A, Norton W, Mitchell S, Jensen R, Jacobsen P, Wilder Smith A. IMPACT Consortium. National Cancer Institute 2018.

[42] Damschroder LJ, Aron DC, Keith RE, Kirsh SR, Alexander JA, Lowery JC (2009). Fostering implementation of health services research findings into practice: a consolidated framework for advancing implementation science. Implement Sci.

[43] CFIR Research Team. Consolidated Framework for Implementation Research 2022 [Available from: https://cfirguide.org/.

[44] Hassett MJ, Cronin C, Tsou TC, Wedge J, Bian J, Dizon DS (2022). eSyM: an electronic health record-integrated patient-reported outcomes-based cancer symptom management program used by six diverse health systems. JCO Clin Cancer Inform.

[45] Hemming K, Haines TP, Chilton PJ, Girling AJ, Lilford RJ (2015). The stepped wedge cluster randomised trial: rationale, design, analysis, and reporting. BMJ..

[46] Hemming K, Taljaard M (2016). Sample size calculations for stepped wedge and cluster randomised trials: a unified approach. J Clin Epidemiol.

[47] Eldridge SM, Costelloe CE, Kahan BC, Lancaster GA, Kerry SM (2016). How big should the pilot study for my cluster randomised trial be?. Stat Methods Med Res.

[48] Campbell MK, Mollison J, Grimshaw JM (2001). Cluster trials in implementation research: estimation of intracluster correlation coefficients and sample size. Stat Med.

[49] Curran GM, Bauer M, Mittman B, Pyne JM, Stetler C (2012). Effectiveness-implementation hybrid designs: combining elements of clinical effectiveness and implementation research to enhance public health impact. Med Care.

[50] Yost KJ, Eton DT, Garcia SF, Cella D (2011). Minimally important differences were estimated for six patient-reported outcomes measurement information system-cancer scales in advanced-stage cancer patients. J Clin Epidemiol.

[51] Chen CX, Kroenke K, Stump TE, Kean J, Carpenter JS, Krebs EE, Bair MJ, Damush TM, Monahan PO (2018). Estimating minimally important differences for the PROMIS pain interference scales: results from 3 randomized clinical trials. Pain..

[52] Cella D, Lai J, Garcia SF, Reeve BB, Weinfurt KP, George J (2008). The patient reported outcomes measurement information system—cancer (PROMIS-Ca): cancer-specific application of a generic fatigue measure. J Clin Oncol.

[53] HealthMeasures. Meaningful change for PROMIS® 2022 [Available from: https://www.healthmeasures.net/score-and-interpret/interpret-scores/promis/meaningful-change.

[54] Analyzing CAHPS Survey Data: agency for healthcare research and quality; 2016 [Available from: https://www.ahrq.gov/cahps/surveys-guidance/helpful-resources/analysis/index.html.

[55] Evensen CT, Yost KJ, Keller S, Arora NK, Frentzel E, Cowans T (2019). Development and testing of the CAHPS cancer care survey. J Oncol Pract.

[56] Boserup B, McKenney M, Elkbuli A (2020). The impact of the COVID-19 pandemic on emergency department visits and patient safety in the United States. Am J Emerg Med.

[57] Schafer JL (1999). Multiple imputation: a primer. Stat Methods Med Res.

